# A case report on pheochromocytoma mimicking as fulminant myocarditis—a diagnostic challenge

**DOI:** 10.3389/fcvm.2024.1326608

**Published:** 2024-03-27

**Authors:** Yanwei Cheng, Ning Ding, Longan Wang, Lijie Qin

**Affiliations:** Department of Emergency, Henan Provincial People’s Hospital, People’s Hospital of Zhengzhou University, People’s Hospital of Henan University, Zhengzhou, China

**Keywords:** pheochromocytoma, fulminant myocarditis, Domperidone, catecholamine-induced cardiomyopathy, alpha-blockade therapy

## Abstract

We present an exceptional case of a 53-year-old female, initially misdiagnosed with fulminant myocarditis, but later correctly diagnosed with pheochromocytoma. The presentation of the patient included a spectrum of symptoms such as headache, chest discomfort, palpitations, and dyspnea, following the intake of Domperidone. Two weeks prior to admission, the patient had experienced episodes of diarrhea and a low-grade fever. Unresolved symptoms and an unmanageable surge in blood pressure despite comprehensive fulminant myocarditis treatment prompted further investigation. The discovery of an adrenal mass via a CT scan and subsequent biochemical tests led to the confirmation of pheochromocytoma. Implementation of alpha-blockade therapy and a successful laparoscopic adrenalectomy resulted in significant clinical improvement. This case underscores the diagnostic intricacies of pheochromocytoma and highlights the need for vigilance when faced with severe, unresponsive cardiovascular symptoms.

## Introduction

Pheochromocytoma, a rare neuroendocrine tumor originating from chromaffin cells of the adrenal medulla, is notorious for its varied clinical presentations, often complicating its diagnosis (1, 2). Myocarditis, an inflammatory condition of the myocardium, has numerous etiologies, encompassing viral infections, autoimmune diseases, and drug reactions (3). It often manifests with symptoms like chest pain, shortness of breath, fatigue, and heart palpitations. In extreme cases, it can progress to heart failure, arrhythmias, or even sudden cardiac death (4). Although distinct, pheochromocytoma can display symptoms akin to myocarditis due to an overproduction of catecholamines, influencing the cardiac function (5). Such overlaps can create confusion, leading to misdiagnosis, particularly in patients with acute cardiac symptoms. We report an unusual case of a 53-year-old woman initially suspected of fulminant myocarditis but ultimately diagnosed with pheochromocytoma. This case aims to bring attention to this potentially fatal condition and offers a thorough review of similar cases documented in literature.

## Case presentation

A 53-year-old female presented to our emergency department urgently with a one-day history of headache, chest tightness, palpitations, and dyspnea. These symptoms emerged around 15 min post Domperidone administration, taken due to a loss of appetite. There was no report of anterior chest pain but she subsequently developed dyspnea and irritability. Remarkably, the patient had endured episodes of diarrhea and low-grade fever two weeks prior to this visit, which spontaneously resolved. The patient's medical history was unremarkable, with no previous episodes of hypertension, diabetes, or coronary heart disease, and no relevant familial history was reported.

**Figure 1:**
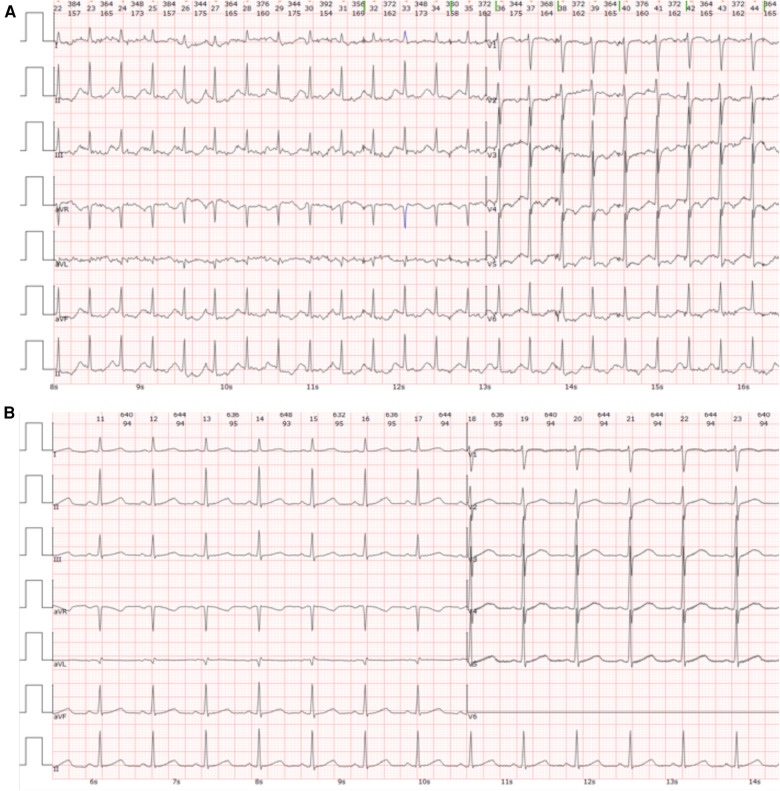
Electrocardiogram (ECG) findings. (**A**) The initial ECG conducted upon admission revealed ST-segment depression in leads II, III, AVF, and V3 to V6, along with T-wave inversions in leads II, AVF, and V4 to V6. (**B**) The ECG taken at discharge exhibited a normal sinus rhythm without significant abnormalities.

**Figure 2:**
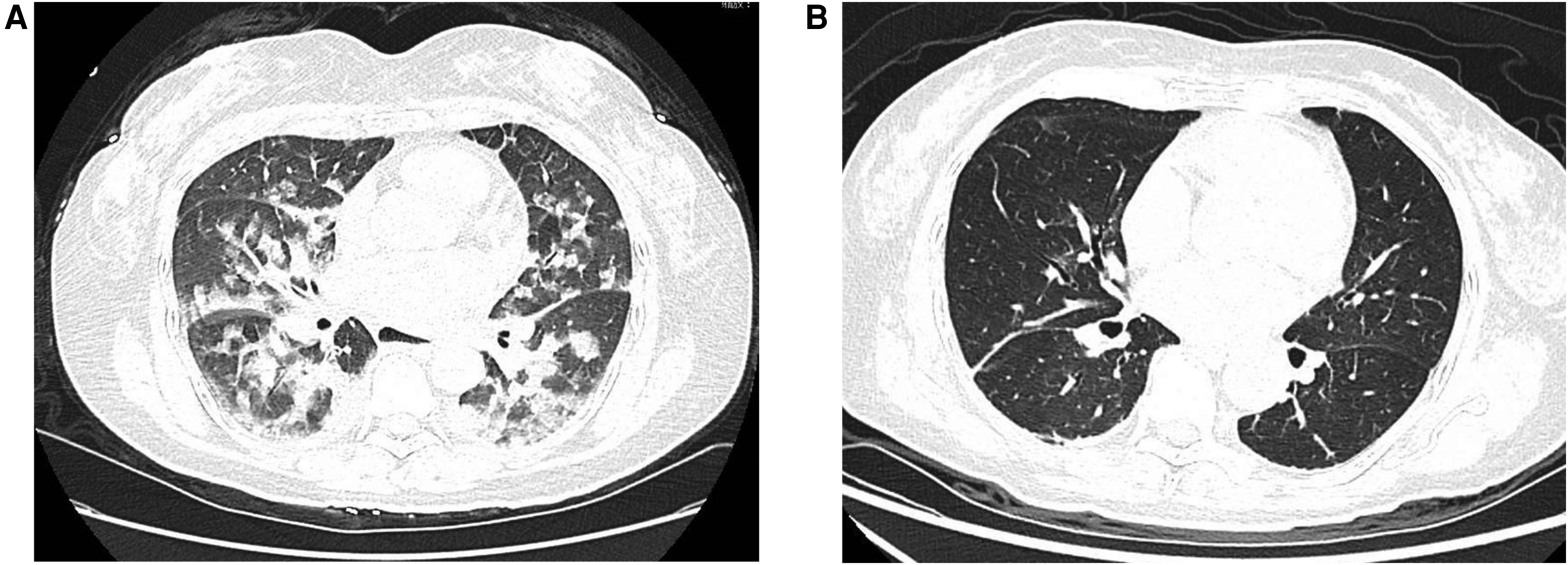
Chest computed tomography (CT) scans. (**A**) The initial chest CT scan performed upon admission demonstrated bilateral pulmonary edema. (**B**) A repeat chest CT scan at discharge showed complete resolution of the previously noted pulmonary edema.

Initial assessment showed a body temperature of 36.5°C, heart rate of 117 beats per minute, respiratory rate of 24 breaths per minute, and blood pressure of 120/74 mmHg. The patient was conscious but exhibited signs of lethargy and pallor. She reported orthopnea and found relief from dyspnea in an upright position. Upon auscultation, crackles were noted in the lower lung fields. The heart rhythm was regular with no noticeable abnormalities. There was no peripheral edema observed. An immediate electrocardiogram (ECG) displayed ST-segment depression in leads II, III, AVF and V3 to V6, as well as T-wave inversions in leads II, AVF and V4 to V6 (Figure 1A). The myocardial enzyme analysis showed significantly elevated levels of myoglobin (MYO, 284.04 ng/ml; normal 0–58 ng/ml), creatine kinase isoenzyme (CK-MB, 100 ng/L; normal 0–5 ng/ml), cardiac troponin-T (cTnT, 1,569 ng/L; normal 0–14 ng/L), as well as N-terminal pro-brain natriuretic peptide (NT-proBNP, 14,058 pg/ml; normal 0–300 pg/ml). Arterial blood gases analysis revealed a pH of 7.24, pO2 of 56 mmHg, pCO2 of 32.7 mmHg, HCO3^−^ of 14 mmol/L, and lactic acid of 8.42 mmol/L. Additional laboratory tests showed leukocytosis, elevated procalcitonin, and liver enzyme abnormalities, while coagulation, electrolyte, and renal function tests remained within normal limits. An echocardiogram showed diffuse left ventricular hypokinesia with a depressed ejection fraction of 26.9% along with mild mitral and tricuspid valve regurgitation. A chest computed tomography (CT) scan suggested bilateral pulmonary edema, potentially compounded by a lung infection (Figure 2A).

**Figure 3:**
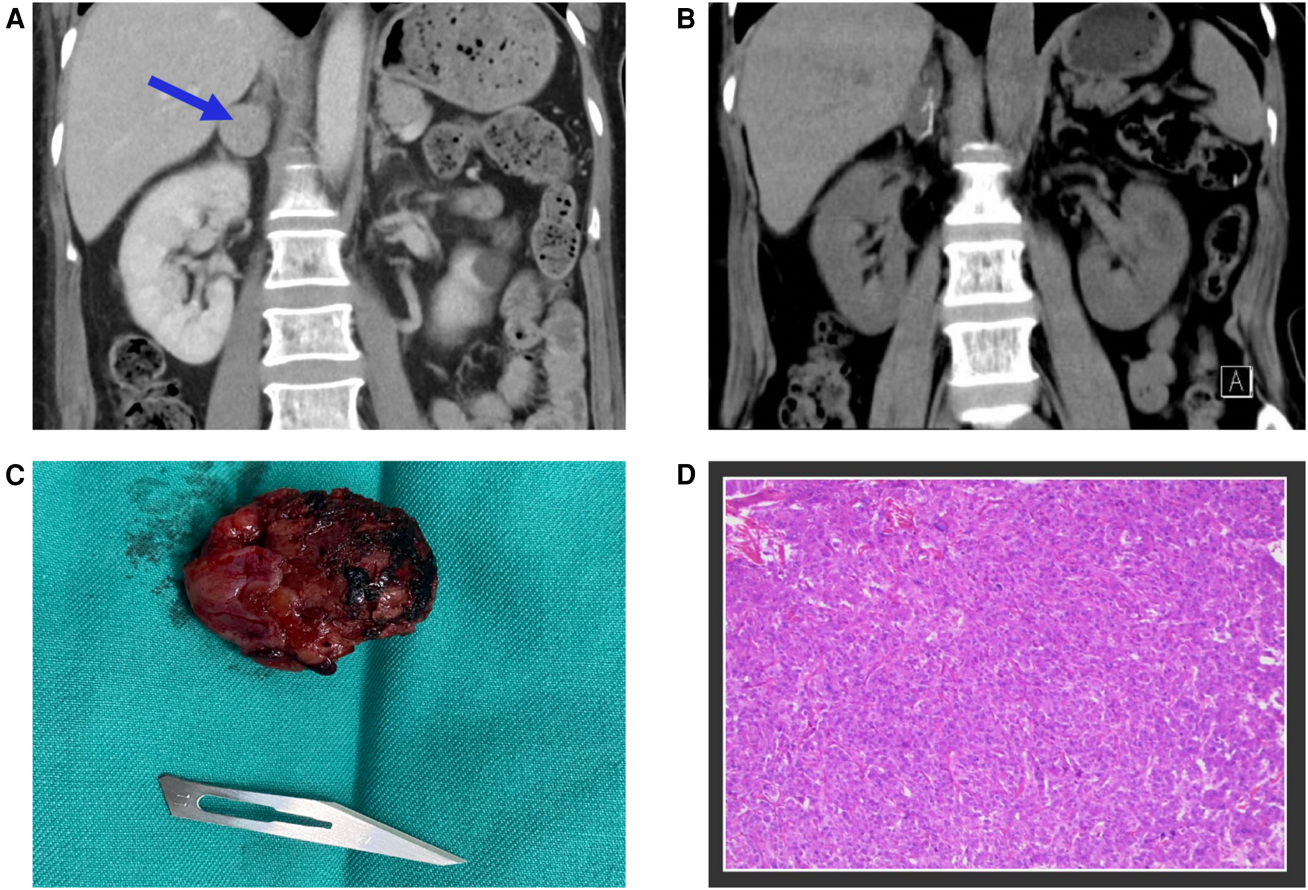
(**A**) The contrast-enhanced abdominal CT scan identified a 26 × 26 × 25 mm mass located in the right adrenal gland, marked with blue arrows. (**B**) The postoperative follow-up CT scan revealed the complete excision of the tumor mass. (**C**) A photograph of the surgically removed tumor during the operation. (**D**) Microscopic examination of the adrenal gland tumor confirmed the diagnosis of pheochromocytoma.

Given the fulminant myocarditis suspicion, the patient was immediately transferred to the emergency intensive care unit (EICU), and a comprehensive treatment strategy was adopted, including non-invasive ventilatory support, antiviral therapy, intravenous immunoglobulin, corticosteroids, anti-infective therapy, and aggressive heart failure management. However, the patient's condition deteriorated, with persistently elevated cardiac markers, and considerations for mechanical circulatory support emerged. On the third day of hospitalization, a sudden and significant increase in blood pressure discouraged the usage of extra-corporeal membrane oxygenation (ECMO) and prompted a reassessment of the initial diagnosis. It is worth noting that despite the administration of medications such as Sodium Nitroprusside and Calcium channel blockers, the patient's blood pressure remained challenging to control. At this point, a contrast-enhanced abdominal CT scan revealed a 26 × 26 × 25 mm mass in the right adrenal gland, leading to suspicion of pheochromocytoma (Figure 3A). This suspicion was supported by elevated plasma metanephrine (2.15 nmol/L; normal < 0.50 nmol/L), plasma normetanephrine (1.24 nmol/L; normal < 0.90 nmol/L), urinary metanephrine (1,954 nmol/24 h; normal < 216 nmol/24 h), and urinary normetanephrine (1,399 nmol/24 h; normal < 312 nmol/24 h). Upon further questioning, the patient stated that she had been having episodic headaches with vomiting and sweating, as well as elevated blood pressure, usually post-Domperidone administration. In light of these findings, the diagnosis was revised from fulminant myocarditis to pheochromocytoma-induced cardiomyopathy, also known as a pheochromocytoma crisis.

Following the initiation of alpha-blockade therapy, the patient's condition gradually improved, with the normalization of various clinical and biochemical markers, improvement in left ventricular ejection fraction (50.8%), and resolution of ECG (Figure 1B) and radiological findings (Figure 2B). Four weeks following alpha-blockade therapy, the patient underwent successful laparoscopic right adrenalectomy (Figures 3B,C), with the pathology report confirming adrenal pheochromocytoma (Figure 3D).

## Discussion

Our report sheds light on the intricate diagnostic conundrums that arise from the diverse presentations of pheochromocytoma, which, in this case, mimicked fulminant myocarditis, leading to a delay in precise diagnosis and intervention.

Pheochromocytoma is an infrequent neuroendocrine tumor originating from the chromaffin cells of the adrenal medulla. Its ability to mimic a multitude of cardiovascular, endocrine, and psychiatric conditions is attributed to its sporadic secretion of catecholamines (6). This attribute warrants its label as “The Great Mimic” (7, 8). Conversely, fulminant myocarditis represents an acute, severe myocardium inflammation, typically induced by viral infections, drugs, or autoimmune diseases. It can escalate swiftly to acute heart failure, shock, and possibly death if not promptly identified and managed (9). A common pathophysiological aspect of both conditions is the cataclysmic surge of catecholamines. Pheochromocytoma provokes an overproduction of catecholamines, potentially leading to catecholamine-induced cardiomyopathy (10). Simultaneously, in fulminant myocarditis, the body's stress response triggers a release of catecholamines, aggravating myocardial injury (11). The coexistence of these clinical features complicates the differentiation of these two conditions, particularly in an acute context.

Our case underscores the cruciality of harboring a high level of suspicion for pheochromocytoma in patients exhibiting severe cardiovascular symptoms. This is particularly significant when the clinical trajectory deviates from the usual course of myocarditis or proves unresponsive to conventional treatment. Clinicians must be alert to signs such as episodic hypertension, fluctuating blood pressure, or intractable hypertension, indicative of possible pheochromocytoma (12). Our case demonstrates that the initial lack of these signs might lead to an erroneous diagnosis, thus accentuating the intricacies in handling such situations. However, a meticulous examination of the patient's history revealed periodic headaches accompanied by vomiting and sweating, and escalated blood pressure post Domperidone intake. This, paired with the subsequent discovery of the adrenal mass, provoked a reconsideration of the initial diagnosis to pheochromocytoma-induced cardiomyopathy or a pheochromocytoma crisis.

The patient consistently experienced identical symptoms post Domperidone intake, prompting our exploration into a potential connection. We hypothesize that the heightened blood pressure and related symptoms observed after Domperidone administration can be attributed to the drug's influence on gastrointestinal motility. Dopamine, a neurotransmitter, naturally restrains smooth muscle contractions in the gastrointestinal tract, playing a pivotal role in motility regulation (13). In contrast, Domperidone interacts with dopamine receptors, effectively blocking dopamine's inhibitory effects (14). This disruption leads to increased smooth muscle activity, thereby amplifying gastrointestinal motility. Considering a potential complication with a pheochromocytoma, a tumor known for excessive catecholamine secretion, the stimulation caused by Domperidone-induced gastrointestinal motility may trigger an abnormal surge in catecholamines. This surge can manifest as periodic episodes of elevated blood pressure, accompanied by symptoms like headache, palpitations, and sweating. This proposed theory aligns seamlessly with the patient's clinical presentation. The symptoms emerged shortly after Domperidone intake, closely mirroring the typical manifestations of pheochromocytoma, including episodic hypertension and other catecholamine-associated symptoms. However, to substantiate this hypothesis and establish a direct causal relationship between Domperidone-induced gastrointestinal motility and pheochromocytoma stimulation, further investigation is essential. Validation of this theory would not only deepen our understanding of the patient's condition but also contribute valuable insights into the potential risks associated with Domperidone use in cases of underlying conditions such as pheochromocytoma.

Diagnostic methods such as biochemical testing and imaging play a crucial role in diagnosing pheochromocytoma, with escalated levels of plasma or urinary metanephrines serving as the principal diagnostic indicator. Moreover, imaging tools like CT scan or magnetic resonance imaging (MRI) aid in identifying adrenal or extra-adrenal tumors (15, 16). The confirmation of a pheochromocytoma diagnosis necessitates initiating alpha-blockade therapy to manage hypertension and prepare the patient for surgical intervention, which is the ultimate treatment strategy for this condition (17). The significant improvement observed in our patient after initiating alpha-blockade and undergoing adrenalectomy reinforces the efficacy of this therapeutic regimen.

An exploration of similar case studies reveals that the singular presentation observed in our case is not an isolated phenomenon. Levin BS et al. presented a case where a patient initially diagnosed with fulminant myocarditis was later discovered to have a pheochromocytoma (18). Likewise, Wu et al. detailed a case involving two middle-aged males with acute myocarditis and cardiogenic shock, necessitating an intra-aortic balloon pump (IABP) and ECMO for life support, ultimately unmasking the presence of a pheochromocytoma (19). In the same vein, Rojbi I et al. documented a case of a 40-year-old female with symptoms suggestive of fulminant myocarditis and COVID-19 pneumonia, later attributed to a pheochromocytoma (20). These cases collectively reiterate the diagnostic hurdles posed by pheochromocytoma mimicking fulminant myocarditis. The capacity of pheochromocytoma to masquerade as other conditions underscores the necessity of considering this uncommon neuroendocrine tumor when assessing patients presenting with severe cardiovascular symptoms.

## Conclusion

In summation, our case accentuates the need for maintaining an expansive differential diagnosis in patients with severe cardiovascular symptoms, even when an initial diagnosis, like fulminant myocarditis, appears feasible. The diagnostic conundrum created by pheochromocytoma, also known as “The Great Mimic”, calls for an elevated level of suspicion, especially when the clinical trajectory strays from the usual path or is unresponsive to conventional therapy. The use of biochemical testing, varied imaging techniques, and an interdisciplinary approach are pivotal for accurate diagnosis and appropriate management. This case serves as an invaluable cue for clinicians to factor in pheochromocytoma in their differential diagnosis, emphasizing the need for further research and heightened awareness of this rare, yet potentially lethal, condition.

## Data Availability

The original contributions presented in the study are included in the article/Supplementary Material, further inquiries can be directed to the corresponding author.
